# Translation, Cultural Adaptation, and Validation of the Swedish Thanatophobia Scale

**DOI:** 10.1177/08258597251388303

**Published:** 2025-11-10

**Authors:** Susanna Pusa, Rebecca Baxter, Anna Sandgren, Sofia Andersson

**Affiliations:** 1 Department of Nursing, 8075 Umeå University, Umeå, Sweden; 2 Center for Collaborative Palliative Care, Department of Health and Caring Sciences, 4180 Linnaeus University, Växjö, Sweden; 3 Regional Cancer Centre North, Norrland University Hospital, Umeå, Sweden

**Keywords:** palliative care, psychometric testing, reliability, thanatophobia, translation, validity

## Abstract

**Objective:**

The aim of this study was to translate, culturally adapt, and validate the Swedish Thanatophobia Scale.

**Methods:**

Forward and back translation, cultural adaptation, content validation, and psychometric testing were undertaken through interviews with experts and surveying of healthcare professionals.

**Results:**

The results from expert ratings (*n* = 7) for the Item Content Validity Index were calculated for scale understandability (mean, 0.90), clarity (mean, 0.91), sensitivity (mean, 0.94), and relevance (mean, 0.96). Interviews (*n* = 10) confirmed the importance of the scale questions and subject matter. The scale was tested among a sample of 386 participants (physicians = 104; registered nurses = 282). No significant differences were found between ratings from physicians and registered nurses. Exploratory Factor Analysis supported the unidimensionality of the scale. The Cronbach's alpha for the total scale was 0.89, indicating good internal consistency.

**Conclusions:**

The Swedish Thanatophobia Scale appears to be a valid and reliable measure of healthcare professionals’ attitudes toward caring for patients in palliative care.

## Introduction

Demand for palliative care is growing in Sweden^1^ and worldwide,^2^ which means that healthcare professionals will increasingly encounter death and dying across a variety of specialties and care settings. Thanatophobia (fear of death and dying) can make it challenging for healthcare professionals to meet patients’ palliative care needs, deliver bad news, and have difficult end-of-life conversations.^3^ Patients with life-threatening illnesses have the right to receive the information they want to know about their diagnosis and illness progression,^4^ and patients and relatives have expressed a desire to receive detailed prognostic information.^5^ However, healthcare professionals have been found to have knowledge deficits in relation to palliative care^6,7^ and are known to be influenced by their own beliefs, philosophies, and fears surrounding death and dying.^8^

Death and dying are essential and inevitable aspects of patient care that impact healthcare professionals in myriad ways. A systematic review exploring the attitudes of nursing professionals toward death revealed that the experience of patient death had a substantial and negative emotional impact on them.^9^ One of the most common strategies used to cope with the death of a patient was avoidance.^9^ Physicians likewise have reported feeling unprepared to undertake palliative care, hesitate when discussing end-of-life care with patients and lack confidence in initiating prognostic discussions, hospice care, and end-of-life care.^10^ A scoping review exploring implementation of palliative care in the intensive care setting likewise identified that healthcare personnel reported challenges in communicating with patients and families about decision-making processes and lack fundamental communication skills when it comes to discussing poor clinical outcomes.^11^ Attitudes toward patients in need of palliative care can serve as barriers or facilitators to palliative care utilization.^12^ Better attitudes toward end-of-life care have been found to be impacted by greater knowledge and perceived self-competence.^13^ Education and clinical experience in palliative care can therefore impact knowledge and beliefs regarding care of patients with palliative care needs.^6^ Aspects that have been found to mitigate the physician's own fear of death include repeated exposure to death and dying, the number of years working as a physician, and the number of years working in palliative care.^8^ Further, education in communication and reflection on fears related to death and dying has resulted in significant reduction in burnout and death anxiety among healthcare professionals working in palliative care settings.^14^ Attitudes toward caring for patients with palliative care needs is thus a relevant phenomenon to explore among healthcare professionals given its impact on patient care.

The Thanatophobia Scale was originally developed to assess death anxiety among medical students working with patients with acquired immunodeficiency syndrome.^15^ This was later adapted for use among healthcare professionals to assess attitudes toward caring for terminally ill persons.^16^ The Thanatophobia Scale has been used among physicians, registered nurses, medical students, and student nurses in different countries and contexts.^16–19^ There is a need for valid and reliable measurements that use common data elements to attitudes toward caring for patients in palliative care among healthcare professionals in Sweden. The aim of this study was to translate, culturally adapt, and validate the Swedish Thanatophobia Scale.

## Methods

### Study Design

This study was performed based on a five-step procedure for instrument translation and adaptation outlined by the World Health Organization.^20^ Permission to translate and validate the Thanatophobia Scale (English version^18^) was obtained from Stephen Mason in March 2020.

### Thanatophobia Scale

The Thanatophobia Scale contains seven items of negative attitudes toward caring for patients in palliative care. Each item is rated on a Likert-type scale ranging from 1 (strongly disagree) to 7 (strongly agree) regarding how strongly the respondent disagrees or agrees with statements expressing negative attitudes. Items are added together to give a sum score that is then divided by the total number of items in the scale (*n* = 7) to give a composite score. Scores above 4 indicate negative attitudes toward caring for patients and their families in palliative care, while scores below 4 indicate positive attitudes.^18^

### Step 1: Forward Translation

First, three independent forward translations of the Thanatophobia Scale were undertaken from English to Swedish. The translators included one man with English as a first language who had worked in the healthcare field in Sweden for 30 years. The other two translators included one woman and one man with Swedish as a first language, good knowledge in English, and no experience working in healthcare. Two of the authors (AS, SA) and two researchers compared the three translations with the original Thanatophobia Scale in English to discuss and resolve any discrepancies.

### Step 2: Back Translation

The back translation to English was performed by two people with English as a first language and good knowledge of Swedish. During this process they did not have access to the original version of the Thanatophobia Scale. Two of the authors (AS, SA) and two researchers then compared the back translations with the Swedish translation and the original Thanatophobia Scale. Some words and meanings had been lost, so the authors (AS, SA) and the two researchers returned to one of the translators to discuss possible interpretations (eg, regarding the word “care”). These interpretations were discussed until consensus was achieved.

### Step 3: Pre-Testing

*Expert ratings*: Experts (*n* = 7) in the area of palliative care (physicians (*n* = 2), registered nurses (*n* = 4), counsellors (*n* = 1)) were recruited from different professional networks during October 2020. The expert group included four women and three men who were professors (*n* = 4) and associate professors (*n* = 3) aged between 59 and 65 years. The experts were asked to rate each of the items in the Swedish Thanatophobia Scale regarding clarity, understanding, relevance, and sensitivity using a Likert-type scale ranging from 1 (not clear, not understandable, not relevant, not sensitive) to 5 (very clear, easy to understand, highly relevant, very sensitive). They were also asked to consider alternative responses or wording for each item and provide an overall reflection regarding the Swedish Thanatophobia Scale.

To assign a value to the expert ratings, the Item Content Validity Index (I-CVI) for each item were calculated.^21^ Before the I-CVI was calculated, the rating domains (understandability, clarity, relevance, and sensitivity) were dichotomized, where ratings of 1 to 3 were combined and assigned a value of 0, and ratings of 4 to 5 were combined and assigned a value of 1. If there are six or more experts, an I-CVI over 0.78 is considered valid.^21^ To assess the probability of a chance occurrence (Pc) regarding the I-CVI for relevance, the following formula was used: [*N*!/*A*!(*N* - *A*)!]*.5*N* (*N* = number of experts; *A* = number of experts agreeing on good relevance). To validate the I-CVI on relevance, a modified Kappa was calculated to provide information about the degree of agreement beyond chance regarding item relevance (I-CVI - Pc)/(1 - Pc). The Kappa ratings were fair (0.40-0.59), good (0.60-0.74), and excellent (>0.75).^21,22^

*Think-aloud interviews*: Ten think-aloud interviews were conducted with physicians (*n* = 3), registered nurses (*n* = 4), and enrolled nurses (*n* = 3). The participants were women (*n* = 6) and men (*n* = 4) with an average age of 55 years (median, 57; range, 34-75). During the interview, participants were shown a first version of the Swedish Thanatophobia Scale and were asked about each item's clarity, understanding, relevance, and sensitivity. The participants were asked to read the item one by one and ‘think aloud’ about whether anything was unclear or if they had concerns about any of the items. Participants were also asked if they had suggestions regarding item formulation or wording. The interviewer asked clarifying questions, such as “Can you tell me more?” and “What do you mean?.” All interviews were audio recorded and analyzed by categorizing responses in relation to the clarity, understanding, relevance, and sensitivity of the scale items.

### Step 4: Cross-Cultural Adaptation

The cross-cultural adaptation of the Swedish Thanatophobia Scale occurred throughout the translation, interview, reconciliation, and evaluation processes. This was to ensure that the Swedish Thanatophobia Scale was suitable for the Swedish healthcare context, both culturally and linguistically, while still maintaining the underlying meanings and concepts of the original version. The authors (AS, SA) and two researchers analyzed the translations, I-CVI responses, and interviews to inform sensitive and responsive cross-cultural adaptations.

### Step 5: Psychometric Testing

Finally, the Swedish Thanatophobia Scale was sent during December 2021 via e-survey to 386 physicians and 1200 registered nurses in all departments at two hospitals to test validity and reliability. Of these, 104 physicians and 282 registered nurses responded. The majority of participants were women (75.1%), with an average age of 45 years (median, 45 years; range, 23-71 years) and an average number of years of experience in their profession of 17.1 years (median, 16 years; range, 6 months to 45 years). An Exploratory Factor Analysis (EFA; maximum likelihood, unrotated) was performed with Eigenvalues >1 and minimum factor loading estimates >0.4.^23^ A scree plot was visually inspected to indicate the number of extractable factors.^24^ Cronbach's alpha, Cronbach's alpha if item deleted, and inter-item correlations were calculated to assess internal consistency and item relevance. Independent samples *t*-tests were used to compare mean differences between physician and registered nurse ratings at item and total scale level. All analyses were undertaken using SPSS (version: 28.0.1.1) and statistical significance was indicated at <.05.

### Ethical Considerations

This study was part of a larger project “Serious Illness Conversations—the Kronoberg Model” and was approved by the Swedish Ethical Review Authority (Dnr 2021-03569).

## Results

### Forward and Back Translation

The forward translation process yielded several linguistic considerations. Some statements required rewording in Swedish to best convey the intended meaning. All changes were made in accordance with the linguistic context of each individual survey item. The back translation indicated minor semantic discrepancies from the original English version, which were discussed and adjusted accordingly. In general, the back translation confirmed there were no differences between the English and Swedish versions. In cases where a direct translation was possible, but not contextually appropriate in the Swedish vernacular, priority was given to understandability and conceptual equivalence. Care was taken to ensure that the intended underlying meaning of the scale remained intact throughout the forward and back translation process.

### Expert Ratings

Two items reported lower CVIs for understandability, item 3 (0.71) and item 4 (0.57) (Table 1). Item 4 also received a lower CVI for clarity and sensitivity (0.71). For this reason, item 4 was rewritten with help of the expert comments. The Pc values for relevance ranged between 0.008 and 0.055. The kappa for all items ranged between 0.85 and 1.0, indicating excellent (>0.74) agreement on content relevance.

**Table 1.** Content Validity Index Ratings and Agreement for Item Understandability, Clarity, Sensitivity, and Relevance.

Item	Agreement Understandability	I-CVI	Agreement Clarity	I-CVI	Agreement Sensitivity	I-CVI	Agreement Relevance	I-CVI
1	6/6	1	5/6	0.83	6/6	1	6/6	1
2	7/7	1	7/7	1	7/7	1	7/7	1
3	5/7	0.71	6/7	0.86	6/7	0.86	6/7	0.86
4	4/7	0.57	5/7	0.71	5/7	0.71	7/7	1
5	7/7	1	7/7	1	7/7	1	6/7	0.86
6	6/6	1	6/6	1	6/6	1	6/6	1
7	7/7	1	7/7	1	7/7	1	7/7	1
I-CVI mean	0.90		0.91		0.94		0.96	

Item Content validity index (I-CVI).

### Think-Aloud Interviews

The 10 participants expressed in the think-aloud interviews that the Swedish Thanatophobia Scale was important and relevant. Although some questions were perceived to contain difficult subject matter, they agreed that the issues were important to discuss and reflect upon. Participants expressed that the questions were formulated “negatively,” but that they understood the purpose of wording the questions in this way. The items in the scale could be perceived as emotional but also provide a sense of safety, as expressed by one participant:

“The first thing I thought (when I saw the questions) was that it's no trouble for me because I work with it (palliative care). But at the same time, if you don’t work with it… it can stir up emotions. It (question 1) felt like a sense of security for me…” (Participant 1)

### Cross-Cultural Adaptation

Following the expert ratings and think-aloud interviews several words/items were identified as being difficult to communicate in practice. To ensure alignment with the underlying content, synonyms were sought for words that were interpreted by the experts as challenging. For example, instead of using the direct translation of the word “helpless” in item 2 (Swedish: hjälplös), the equivalent word for “inadequate/insufficient” was used (Swedish: otillräcklig). Likewise, using the direct translation of item 5 (managing dying patients traumatizes me) was found to be problematic by the experts as the word traumatize (Swedish: traumatiserar) was perceived to be harsh and judgmental and is not used in relation to patient care in Sweden. Instead, the item was rewritten to express “taking care of dying patients affects me negatively.” Cross-cultural considerations were also made in relation to the Swedish healthcare context. For example, inclusion of the term “ward” in an item was perceived to limit the meaning to the hospital context, but in Sweden palliative care can occur in a variety of contexts (eg, hospital, home care, hospice). For this reason, “from my ward” was removed from item 2 in the Swedish version. Table 2 provides an overview of the semantic discrepancies and resolutions that emerged following the translation, expert review, and cross-cultural adaptation process.

**Table 2.** Semantic Discrepancies and Adapted Terms.

Item	Original Term (English)	Adapted Term (Swedish/*English*)	Revision Stage
1	Uneasy	Obekväm (*Uncomfortable*)	Forward and back translation
2	Helpless	Otillräcklig (*Inadequate/insufficient*)	Expert ratings and think-aloud interviews
3	Frustrating	Påfrestande (*Stressful/strenuous*)	Expert ratings and think-aloud interviews
4	Managing	Att vårda (*Caring for/treating*)	Forward and back translation
4	Traumatizes	Påverkar mig negativt (*Affects me negatively*)	Expert ratings
5	It makes me uncomfortable…	Jag känner mig obekväm… (*I feel uncomfortable…*)	Expert ratings and think-aloud interviews
6	I don’t look forward to…	Jag önskar inte… (*I don’t wish to…*)	Expert ratings and think-aloud interviews
6	Being the personal physician	Vara ansvarig läkare/sjuksköterska (*Being the responsible physician/ nurse*)	Forward and back translation

### Psychometric Testing

The Kaiser–Meyer–Olkin measure (0.905) verified sampling adequacy and Bartlett's Test of Sphericity (Chi-square 1224.51; df, 21; *p* < .001) indicated that correlations between items were large enough to support EFA. The item-level correlation matrix is presented in Supplementary File S1. The scree plot inflection flattened after the first factor, indicating that additional factors contributed minimal variance. One factor was extracted (loading range: 0.653-0.788) which accounted for 53.16% of the total variance. The full factor matrix is presented in Supplementary File S2. The Cronbach's alpha for the Swedish Thanatophobia Scale was 0.886 (*n* = 376). The Cronbach's alpha if item deleted did not increase for any of the items (range, 0.862-0.879). The Cronbach's alpha was 0.85 among the physicians and 0.89 among the registered nurses. The inter-item correlations for all ratings ranged from 0.392 to 0.610 (physicians, 0.255-0.677; registered nurses, 0.407-0.633). There were no significant differences in mean item scores (Table 3), nor in mean scores for the total scale (*p* = .268; CI lower −0.107, upper 0.381) between registered nurses and physicians.

**Table 3.** Comparison of Physician and Registered Nurse Ratings.

Item	Rating	Physicians	Registered Nurses	*p*-Value^*^ (95% CI Lower/Upper)
1	Total	104	281	.514 (−0.442/0.222)
	Mean (SD)	2.45 (1.36)	2.34 (1.51)	
	Median	2.00	2.00	
	Min-max	1-7	1-7	
	1Q-3Q	2-3	1-3	
2	Total	104	281	.892 (−0.388/0.338)
	Mean (SD)	3.10 (1.50)	3.07 (1.65)	
	Median	3.00	3.00	
	Min-max	1-7	1-7	
	1Q-3Q	2-4	2-4	
3	Total	103	280	.451 (−0.220/0.494)
	Mean (SD)	2.50 (1.53)	2.63 (1.59)	
	Median	2.00	2.00	
	Min-max	1-7	1-7	
	1Q-3Q	1-3	1-4	
4	Total	103	280	.767 (−0.392/0.289)
	Mean (SD)	2.57 (1.45)	2.52 (1.52)	
	Median	2.00	2.00	
	Min-max	1-7	1-7	
	1Q-3Q	2-3	1-4	
5	Total	101	279	.187 (−0.129/0.655)
	Mean (SD)	2.44 (1.56)	2.70 (1.77)	
	Median	2.00	2.00	
	Min-max	1-7	1-7	
	1Q-3Q	1-3	1-4	
6	Total	103	281	.303 (−0.167/0.535)
	Mean (SD)	1.88 (1.50)	2.07 (1.57)	
	Median	1.00	1.00	
	Min-max	1-7	1-7	
	1Q-3Q	1-2	1-2	
7	Total	104	281	.228 (−0.123/0.516)
	Mean (SD)	1.97 (1.29)	2.17 (1.46)	
	Median	2.00	2.00	
	Min-max	1-7	1-7	
	1Q-3Q	1-2	1-3	

* Independent samples *t*-test.

Abbreviations: SD, standard deviation; Min-max, minimum-maximum; 1Q-3Q, interquartile range.

## Discussion

This study aimed to translate, culturally adapt, and validate the Swedish Thanatophobia Scale. The translation process from English to Swedish, and the associated cross-cultural adaptations, resulted in several linguistic modifications. Each modification was considered in relation to single words, the item as a whole, as well as underlying meanings to ensure equivalence. The Thanatophobia Scale has been adjusted to fit the Swedish healthcare context by, for example, using wording that reflects the broad setting of palliative care (ie, inpatient and outpatient care) and the personnel who work within it (ie, physicians and registered nurses). In this way, the expert ratings and think-aloud interviews both enhanced and confirmed the usability of the Thanatophobia Scale for the Swedish healthcare context.

The Thanatophobia Scale has been primarily used to evaluate undergraduate medical and nursing education in different countries.^25–27^ Adapting the Thanatophobia Scale to the Swedish context to focus on practicing physicians and registered nurses is important as both professions regularly encounter death in their clinical practice. Fear of death may influence healthcare professionals’ interactions, decision-making, and ability to offer compassionate care.^28,29^ However, the Thanatophobia Scale has only been validated for use among nurses in one Turkish study.^30^ The findings from the present study showed no significant differences between the scores from physicians and registered nurses, as both scored in the lower range of the scale for all questions. These findings suggest that the Swedish Thanatophobia Scale can be used among both professions, reinforcing its relevance for assessing attitudes toward caring for patients in palliative care among healthcare providers in Sweden.

Regarding validity, accurate translation of wording from the language of origin is important to maintain the integrity of the scale. When translating and culturally adapting the Thanatophobia Scale it was necessary to ensure that the statements conveyed equivalent meanings in the target culture. The reliability of the translation method used in this study is strengthened as it followed established guidelines.^20^ The experts rated the majority of items highly on the CVI, indicating high internal consistency (>0.78). The think-aloud interviewees confirmed the applicability of the content and the appropriateness of the translation for the Swedish context.

The EFA confirmed the unidimentionality of the Swedish Thanatophobia Scale, consistent with other studies.^16,18^ The Cronbach's alpha of 0.89 is indicative of high internal consistency with low item redundancy. In the original scale development, Cronbach's alpha for the scale was 0.82 to 0.87 for practicing physicians, student nurses, and medical students.^16^ This is similar to other studies using Thanatophobia Scale for nurses in Turkey 0.85,^30^ 0.87,^31^ 0.83,^32^ 0.90^33^; in Brasil for medical students 0.82^34^; in Korea for clinical nurses 0.92^35^; and in the United Kingdom for medical students 0.84.^18^ The findings from the present study indicate that the Swedish Thanatophobia Scale can be reliably used among different professional groups, supporting its robustness as a measure of attitudes toward caring for patients in palliative care in the Swedish healthcare setting.

### Limitations

The study exhibited a low response rate for the psychometric testing (physicians, 26.9%; registered nurses, 23.5%), which may be attributable to the fact that the survey was conducted online. This phenomenon is not unusual when surveying healthcare workers using digital survey tools.^36,37^ Another possible reason for the low response rate may be that the survey was sent to all departments at two hospitals. Non-participants may not have been regularly involved in palliative care of patients, and the healthcare professionals who did participate were not asked about how often they cared for patients with palliative care needs. Future studies may consider further testing of the scale with this in mind. Stability was not evaluated in this study, future studies are therefore required to establish the test-retest reliability of the Swedish Thanatophobia Scale.

### Clinical Implications

The Swedish Thanatophobia Scale demonstrates validity and reliability for assessing attitudes toward caring for patients in palliative care among physicians and nurses. To further establish its applicability, future research should explore its use in diverse study populations and clinical settings. Integrating the scale into Swedish nursing and medical education and professional development programs may provide valuable insights into how these attitudes influence care practices. A deeper understanding of attitudes toward caring for patients in palliative care among healthcare professionals could ultimately support identification of strategies to improve palliative care and enhance patient outcomes.

## Conclusions

The Swedish Thanatophobia Scale has been shown to be a reliable and valid instrument for measuring attitudes toward caring for patients in palliative care among physicians and nurses. The scale can serve as a useful tool for future assessments and evaluations of thanatophobia in education and the Swedish healthcare context.

## Supplemental Material

sj-docx-1-pal-10.1177_08258597251388303 - Supplemental material for Translation, Cultural Adaptation, and Validation of the Swedish Thanatophobia Scale Supplemental material, sj-docx-1-pal-10.1177_08258597251388303 for Translation, Cultural Adaptation, and Validation of the Swedish Thanatophobia Scale by Susanna Pusa, Rebecca Baxter, Anna Sandgren and Sofia Andersson in Journal of Palliative Care

sj-docx-2-pal-10.1177_08258597251388303 - Supplemental material for Translation, Cultural Adaptation, and Validation of the Swedish Thanatophobia Scale Supplemental material, sj-docx-2-pal-10.1177_08258597251388303 for Translation, Cultural Adaptation, and Validation of the Swedish Thanatophobia Scale by Susanna Pusa, Rebecca Baxter, Anna Sandgren and Sofia Andersson in Journal of Palliative Care
