# Supplementary material for: Translation, Cultural Adaptation, and Validation of the Swedish Thanatophobia Scale
Source: J Palliat Care. 2025 Nov 10;41(3):314–21. doi: 10.1177/08258597251388303 (PMC13234302; doi:10.1177/08258597251388303)
Supplement: sj-docx-1-pal-10.1177_08258597251388303 - Supplemental material for Translation, Cultural Adaptation, and Validation of the Swedish Thanatophobia Scale [file sj-docx-1-pal-10.1177_08258597251388303.docx]

**Supplementary File 1**

Correlation matrix for the Swedish Thanatophobia Scale

| **Item** | **1** | **2** | **3** | **4** | **5** | **6** | **7** |
| --- | --- | --- | --- | --- | --- | --- | --- |
| **1** | 1.000 | .610 | .565 | .597 | .566 | .525 | .557 |
| **2** | .610 | 1.000 | .500 | .494 | .428 | .392 | .438 |
| **3** | .565 | .500 | 1.000 | .589 | .545 | .525 | .531 |
| **4** | .597 | .494 | .589 | 1.000 | .544 | .582 | .468 |
| **5** | .566 | .428 | .545 | .544 | 1.000 | .546 | .604 |
| **6** | .525 | .392 | .525 | .582 | .546 | 1.000 | .506 |
| **7** | .557 | .438 | .531 | .468 | .604 | .506 | 1.000 |
