# Supplementary material for: Translation, Cultural Adaptation, and Validation of the Swedish Thanatophobia Scale
Source: J Palliat Care. 2025 Nov 10;41(3):314–21. doi: 10.1177/08258597251388303 (PMC13234302; doi:10.1177/08258597251388303)
Supplement: sj-docx-2-pal-10.1177_08258597251388303 - Supplemental material for Translation, Cultural Adaptation, and Validation of the Swedish Thanatophobia Scale [file sj-docx-2-pal-10.1177_08258597251388303.docx]

**Supplementary File 2**

Factor matrix for the Swedish Thanatophobia Scale

| **Item** | **Factor 1** |
| --- | --- |
| **1** | .788 |
| **2** | .653 |
| **3** | .746 |
| **4** | .755 |
| **5** | .742 |
| **6** | .704 |
| **7** | .708 |

^Note: 1 factor extracted accounting for 53.16% of the total variance, 3 iterations required.^
